# Optimal bone-implant contact sites in the zygomatic region for quad zygomatic implants placement: a retrospective study in Vietnamese patients on CBCT

**DOI:** 10.1038/s41405-025-00350-8

**Published:** 2025-06-20

**Authors:** Mi Nguyen-Tra Le, Tri Minh Tran, Phuc Ngoc Nguyen, Hung Chi Vo, Lam Hung Tran

**Affiliations:** 1 Faculty of Dentistry, University of Medicine and Pharmacy at Ho Chi Minh City, Ho Chi Minh City, Vietnam; 2 Worldwide Hospital, Ho Chi Minh City, Vietnam; 3 Faculty of Dentistry, Van Lang University, Ho Chi Minh City, Vietnam

**Keywords:** Oral anatomy, Dental treatments

## Abstract

**Introduction:**

Zygomatic implants represent a reliable treatment modality for patients with severe maxillary bone resorption, eliminating the need for bone grafting and enabling immediate loading. This study utilized cone beam computed tomography (CBCT) to identify optimal zygomatic bone regions for implant placement by assessing bone-implant contact (BIC) while minimizing intrusion risks into the infratemporal fossa (ITF). Additionally, differences in zygomatic characteristics between males and females were investigated to address the limited evidence regarding the influence of biological sex on BIC and implant stability.

**Methods:**

This retrospective study analyzed CBCT scans from 20 fully edentulous patients (9 male and 11 female) with severe maxillary resorption. Zygomatic bone thickness, length, and BIC were measured at 12 anatomical points across the superior, middle, and inferior regions using standardized CBCT imaging and Nobel Clinician software. Virtual implants were placed to evaluate intrusion into the infratemporal fossa. Statistical analyses, including Kruskal-Wallis and Mann–Whitney U tests, were conducted to compare zygomatic measurements across regions and between genders.

**Results:**

The greatest bone thicknesses in the superior, middle, and inferior regions were observed at Point A_1_ (8.53 ± 1.63 mm), Point B_1_ (6.97 ± 1.01 mm), and Point C_0_ (6.36 ± 1.02 mm), respectively. Point A_3_ (17.65 ± 2.24 mm) in the anterior region and Point B_1_ (13.34 ± 2.35 mm) in the posterior region were identified as optimal implant sites, providing the highest BICs while minimizing intrusion risks. Zygomatic thickness and BIC at these optimal sites were significantly greater in males than females (*p* < 0.01).

**Conclusion:**

Point A_3_ and Point B_1_ are the most suitable sites for zygomatic implant placement. Quad zygomatic implants may achieve enhanced primary stability in males than in females due to greater zygomatic bone thickness and BIC.

## Introduction

Dental implant placement has become an increasingly popular solution for tooth restoration, offering patients long-term functional and esthetic benefits. However, in cases of severe maxillary bone resorption, conventional implant placement is often not feasible, posing challenges for oral rehabilitation. The zygomatic bone, with its dense quality, large surface area, and sufficient volume, provides a reliable alternative for implant anchorage [1–3]. Zygomatic implants offer several advantages over traditional implants, including the elimination of bone grafting procedures and reduced surgical and recovery times [4–6]. Long-term studies report high survival rates for zygomatic implants, ranging from 93.8 to 100%, as demonstrated by Felice et al. [3], Malo et al., Davo et al., and Wang et al. [3, 7–9]. In cases of severe resorption, placement of four zygomatic implants (quad-zygoma technique) is widely recommended to achieve sufficient prosthetic support [10]. Aboul-Hosn Centenero et al. [11]. reported comparable survival rates between the quad-zygoma technique and hybrid approaches using both zygomatic and conventional implants [11]. Accurate assessment of zygomatic bone dimensions is essential for optimizing implant positioning, maximizing bone-implant contact (BIC), and minimizing the risk of complications, particularly intrusion into the infratemporal fossa (ITF).

BIC within the zygomatic bone is a critical factor for predicting implant osseointegration, primary stability, and long-term success. Greater BIC enhances implant stability and bone integration, particularly critical in the compromised bone quality of the atrophic maxilla. Romeed et al. [12] recommended a minimum zygomatic implant length of 15 mm, noting significantly increased stress concentrations when implant length is reduced to 10 mm [12]. While previous studies have assessed the maximum zygomatic bone thickness on cadavers for optimal implant apex placement, few have evaluated BIC directly. Hung et al. [13]. used cone beam computed tomography (CBCT) to identify zygomatic bone regions providing the highest BIC while avoiding intrusion complications for quad zygomatic implant placement [13]. Although BIC is traditionally considered a histological measure reflecting the percentage of bone in direct contact with an implant surface, CBCT-based simulations allow for a radiographic surrogate measurement that aids in preoperative planning and the identification of anatomically favorable implant sites. This radiographic BIC evaluation informs several critical aspects of surgical planning. First, it helps identify optimal implant entry and exit points along the zygomatic trajectory, ensuring the implant path engages the greatest volume of bone. Second, it supports selection of implant length and angulation tailored to the patient’s anatomy, particularly important in cases with asymmetric or limited bone volume. Third, by visualizing areas of low BIC or potential cortical perforation, clinicians can proactively adjust their surgical plan to avoid complications, such as implant instability or penetration into adjacent anatomical spaces. Finally, BIC mapping can improve prosthetic outcomes by enhancing implant distribution and load-bearing capacity. Hung et al. also reported significant differences in zygomatic bone length and thickness between sexes; however, the influence of biological sex on BIC remains unclear, leaving a gap in knowledge regarding its potential impact on implant stability.

In this study, CBCT imaging was used to evaluate fully edentulous Vietnamese patients. The specific aims of the study were to: (1) describe zygomatic bone thickness and length; (2) to identify regions with the highest BIC while minimizing intrusion risks; and (3) to compare zygomatic bone measurements and BIC values between male and female groups.

## Materials and methods

### Study design/sample

This retrospective study was approved by the Ethics Committee of the University of Medicine and Pharmacy at Ho Chi Minh City (No. 632/HDDD-DHYD). Patients with CBCT scans confirming complete maxillary edentulism and severe alveolar ridge resorption, unsuitable for conventional implants, were recruited from March 1^st^, 2019, to September 30^th^, 2024. This study ensures complete anonymity of the collected data, as no personally identifiable information was recorded.

The inclusion criteria included (1) patients with complete maxillary edentulism who have received four zygomatic implants, and (2) patients classified with bone resorption types 4 and 5 according to the Cawood and Howell classification [14].

The exclusion criteria included (1) patients with tooth loss from maxillofacial trauma or segmental jaw resection, and (2) patients with congenital/acquired zygomatic or maxillary bone abnormalities.

### Data collection

CBCT scans were acquired using the ICAT 3D Imaging system (Imaging Sciences International, Hatfield, PA, USA) with standardized imaging parameters: tube current of 5 mA, tube voltage of 120 kV, maximum resolution of 0.25 mm pixel size, field of view (FOV) of 25 cm (diameter) × 17 cm (height), and a scan duration of 16–20 s. The scanned region included the maxilla. CBCT data were imported into Nobel Clinician software for image analysis and measurements.

Image analysis was performed following protocol by Hung et al. [13]. (Figs. S1, S2). Briefly, The IM line connected the lowest points on the infraorbital margin, while the LM line passed through the most lateral point on the orbital margin (Fig. S1A). Their intersection defined Point C, with Point O located at the angular bisector of these lines (Fig. S1B). Measurement lines L_0_ to L_3_ were drawn parallel to L_1_ (connecting Points C and O) at 5 mm intervals, extending from the orbital margin to the inferior border of the zygoma, representing the potential apical region for zygomatic implants (Fig. S1B, C). Lines L_0_ to L_3_ were subdivided into six anatomical points (A_0_, B_0_, C_0_, A_3_, B_3_, C_3_) across superior, middle, and inferior regions. Points E_1_ and E_2_ were defined along the alveolar crest to guide anterior and posterior implant placement (Fig. S1D). Zygomatic bone thickness was measured at 12 points, with bone length assessed along L_0_–L_3_ (Fig. S2A). Virtual implants (4 mm in diameter, NobelZygoma, Nobel Biocare, Sweden) were placed using planning software, with BIC values calculated at both facial and temporal aspects (Fig. S2B). Implant apices were positioned at defined points (A_0_–A_3_, B_0_–B_3_, C_0_–C_3_), and intrusion depth into ITF was recorded by measuring the linear distance from the outer cortex of the zygomatic bone to the implant surface if it extends beyond the bone (Fig. S2C). Bilateral measurements ensured a comprehensive evaluation of zygomatic anatomy and implant positioning.

### Data analysis

Descriptive analyses were conducted to present the demographic profile of the study population. Zygomatic bone thickness and length were compared among regional groups using the Kruskal–Wallis test, and between sexes using the Mann–Whitney U test. Statistical analyses were conducted using GraphPad Prism v.10.4.1 (GraphPad Software Inc., San Diego, CA, USA). A *p* value of <0.05 was considered statistically significant.

## Results

### Patients’ demographics

The study involved 20 edentulous patients (male = 9, female = 11) with the mean age of 59.7 ± 8.3 years, ranged from 43 to 71 years. Fifty five percent of the patients were above 60 years old.

### The zygomatic lengths at lines and thicknesses at points

**Fig. 1:**
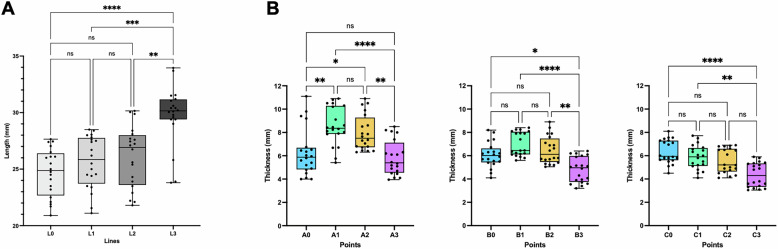
Zygomatic bone lengths. Zygomatic bone lengths at lines from line 0 to line 3 (**A**) and thicknesses at points on the superior, middle, and inferior areas (**B**). Comparisons of the mean lengths among lines or thicknesses among points were analyzed with a Kruskal-Wallis test. ns, not significant; *, *p* < 0.05; **, *p* < 0.01, ***, *p* < 0.001, ****, *p* < 0.0001.

Among the 40 zygomatic bones from 20 patients, the mean zygomatic lengths at different lines progressively increased from L_0_ (24.69 ± 2.12 mm) to L_3_ (29.77 ± 2.63 mm). Notably, L_3_ was significantly longer than any other line, whereas no significant pairwise differences were observed among L_0_, L_1_, and L_2_ (Fig. 1A).

The greatest zygomatic thickness in the superior, middle, and inferior regions was observed at Point A_1_ (8.53 ± 1.63 mm), Point B_1_ (6.97 ± 1.01 mm), and Point C_0_ (6.36 ± 1.02 mm), respectively (Fig. 1B). In the superior and middle regions, thickness gradually decreased from Points A_1_/B_1_ toward both anterior (A0/B0) and posterior (A_3_/B_3_) points. In the inferior region, thickness declined from Point C_0_ toward the posterior point (C_3_) (Fig. 1B).

Interestingly, the variation in thickness among points was more pronounced in the superior region (A points) compared to the middle (B points) and inferior regions (C points). Specifically, the thickness at Point A_1_ was significantly higher than at both Point A_0_ and A_3_ (*p* = 0.0014 and *p* < 0.0001, respectively). Meanwhile, the thickness at Point B_1_ was significantly higher than at only Point B_3_ (*p* < 0.0001), and thickness at Point C_0_ was significantly higher than at only Point C_3_ (*p* < 0.0001).

### Zygomatic thickness and length in correlation with sex and age

**Fig. 2:**
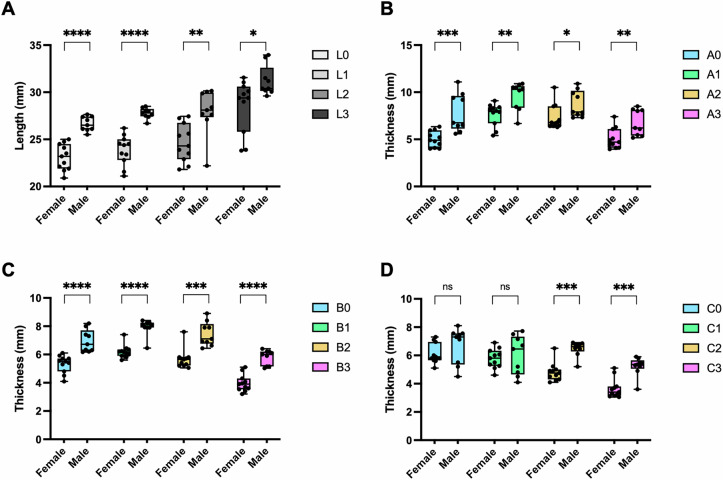
Comparison of the zygomatic lengths. Comparison of the zygomatic lengths at lines (**A**) and thickness at points (**B**–**D**) between male and female. Comparisons of the mean values between male and female were analyzed with a Mann–Whitney U test. ns, not significant; *, *p* < 0.05; **, *p* < 0.01, ***, *p* < 0.001, ****, *p* < 0.0001.

Comparison of the zygomatic length between males and females revealed that all measured zygomatic lines were significantly longer in males than in females (Fig. 2A). Regarding zygomatic thickness, all points, except points C_0_ and C_1_, were significantly thicker in males than in females (Fig. 2B–D). In contrast, no significant differences in zygomatic bone thickness or length were observed between individuals under 60 and those over 60 years old.

### The zygomatic BICs of the virtual implants and intrusion in ITF at different points

Virtual implants were placed on 40 zygomatic bones of 20 edentulous patients, with the apical points of the mesial implant were sequentially positioned from Point A_0_ to A_3_, while those of the distal implant were arranged from Point B_0_ to B_3_ and Point C_0_ to C_3_.

**Fig. 3:**
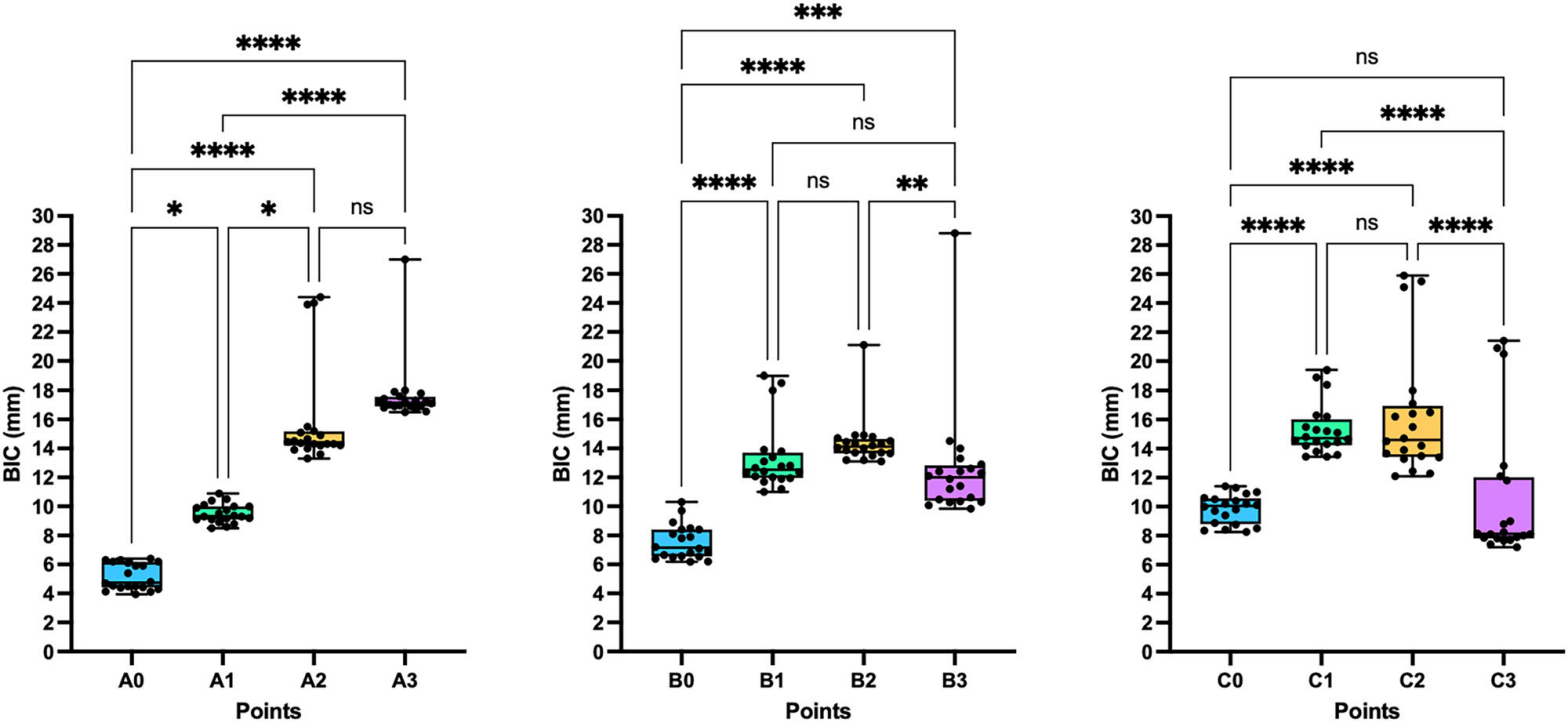
The zygomatic BICs of the virtual implants at different points. Comparisons of the mean BIC among different points in the superior, middle, and inferior regions were analyzed with a Kruskal-Wallis test. ns, not significant; *, *p* < 0.05; **, *p* < 0.01, ***, *p* < 0.001, ****, *p* < 0.0001.

In the superior region, Point A_3_ (17.65 ± 2.24 mm) exhibited the highest zygomatic BIC, with values gradually decreasing from Point A_3_ to Point A_0_ (Fig. 3). In the middle and inferior regions, the highest zygomatic BICs were observed at Point B_2_ (14.41 ± 1.67 mm) and Point C_2_ (16.21 ± 4.33 mm), respectively. The BIC values subsequently decreased from Point B_2_ to the posterior point (Point B_3_, *p* < 0.01) and the anterior point (Point B_0_, *p* < 0.0001), as well as from Point C_2_ to the posterior point (Point C_3_, *p* < 0.0001) and the anterior point (Point C_0_, *p* < 0.0001).

Regarding the intrusion into the ITF, no intrusion was observed in the superior region (Points A_0_–A_3_) following zygomatic implant placement (Table 1). In the middle region, implantation at Point B_3_ had the highest incidence of ITF intrusion (90%), followed by Point B_2_ (30%), with average intrusion depths of 2.13 ± 0.96 mm and 0.67 ± 0.94 mm, respectively. No intrusion complications were observed at Points B_1_ and B_0_. In the inferior region, implantation at Point C_3_ resulted in the highest incidence of ITF intrusion (100%), followed by Point C_2_ (85%) and Point C_1_ (20%), with average intrusion depths of 4.06 ± 1.18 mm, 2.37 ± 1.38 mm, and 0.32 ± 0.58 mm, respectively. No intrusion complications were observed at Point C_0_. The difference in intrusion depth among the four B points and among the four C points was statistically significant (*p* < 0.0001, Table 1).

**Table 1:** The intrusion rate and depth of implant intruded into infratemporal fossa.

Region	Apical point	Intrusion rate into ITF (%)	Intrusive depth into ITF (mm)	*p* value^a^
**Superior**	**A**_**0**_	0	0	N/A^b^
	**A**_**1**_	0	0	
	**A**_**2**_	0	0	
	**A**_**3**_	0	0	
**Middle**	**B**_**0**_	0	0	<0.0001
	**B**_**1**_	0	0	
	**B**_**2**_	30	0.67 ± 0.94	
	**B**_**3**_	90	2.13 ± 0.96	
**Inferior**	**C**_**0**_	0	0	<0.0001
	**C**_**1**_	20	0.32 ± 0.58	
	**C**_**2**_	85	2.37 ± 1.38	
	**C**_**3**_	100	4.06 ± 1.18	

^a^Kruskal – Wallis test.

^b^Not applicable.

### BICs of the virtual implants in correlation with sex

**Fig. 4:**
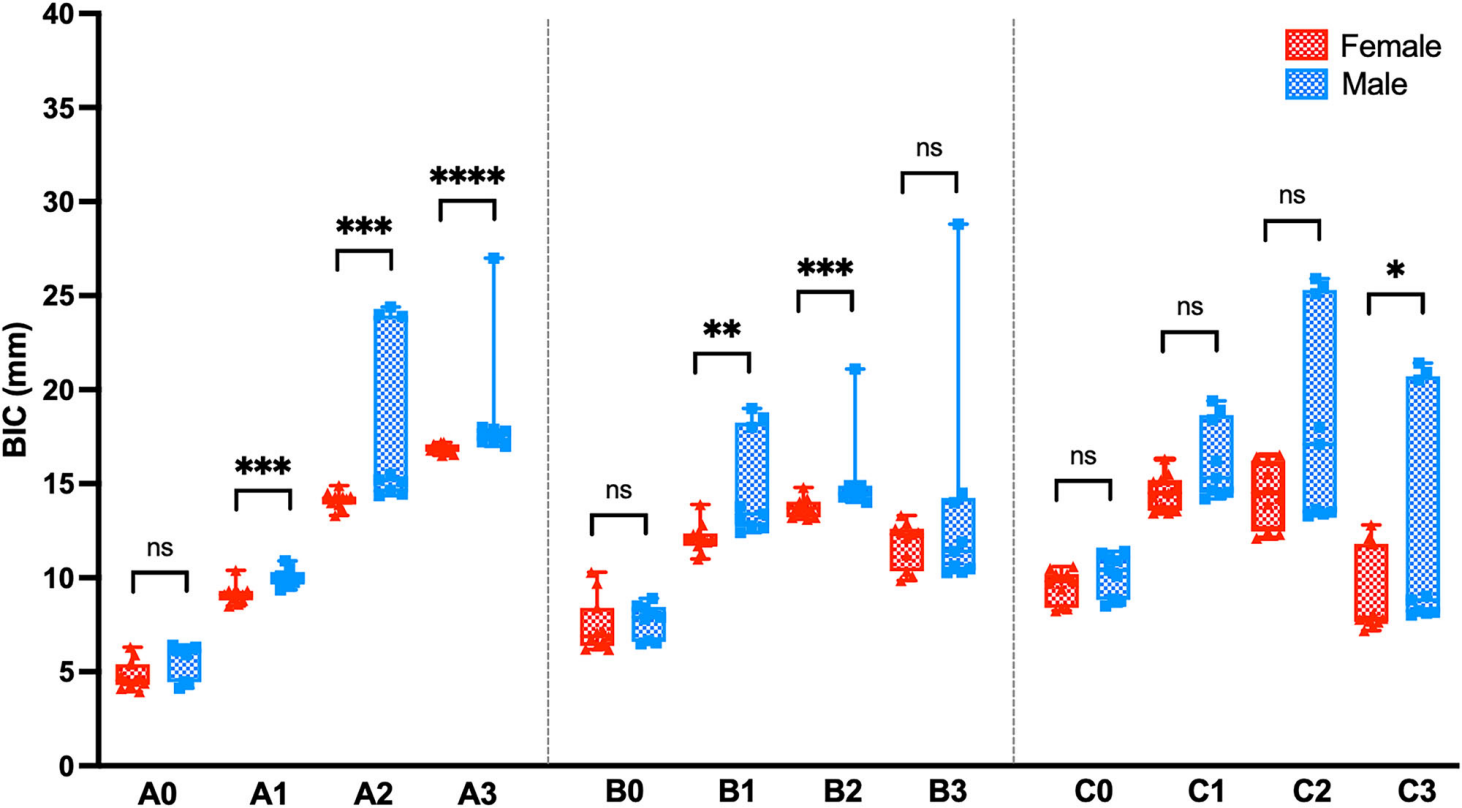
The zygomatic BICs of the virtual implants at different points by sex. Comparisons of the mean BIC at different points between male and female were analyzed with a Mann–Whitney U test. ns, not significant; *, *p* < 0.05; **, *p* < 0.01, ***, *p* < 0.001, ****, *p* < 0.0001.

Given the observed differences in zygomatic length and thickness between males and females, we also compared the BICs of virtual implants between the two groups. The data indicated that the BIC patterns were similar in both sexes, with Points A_3_, B_2_, and C_2_ exhibiting the highest BICs in the superior, middle, and inferior regions, respectively (Fig. 4). However, half of the analyzed points, including Points A_1_, A_2_, A_3_, B_1_, B_2_, and C_3_, demonstrated significantly higher BICs in males than in females (Fig. 4).

At point A_3_—the site with the highest BIC and no risk of intrusion in the anterior region—both zygomatic bone thickness and the BIC of the virtual implant were significantly greater in males than in females (*p* = 0.0045 and *p* < 0.0001, respectively, Table 2). Similarly, at point B_1_—the corresponding optimal site in the posterior region—males also exhibited significantly greater zygomatic thickness and BIC values compared to females (*p* < 0.0001 and *p* = 0.0012, respectively, Table 2).

**Table 2:** Comparison of the zygomatic thickness and BICs of virtual implants at Point A_3_ and B_1_ between male and female.

Points	Male	Female	*p* value^a^
**A**_**3**_			
Zygomatic thickness	6.78 ± 1.38	5.05 ± 1.09	0.0045
BIC of virtual implant	18.58 ± 3.17	16.88 ± 0.24	< 0.0001
**B**_**1**_			
Zygomatic thickness	7.91 ± 0.57	6.19 ± 0.46	< 0.0001
BIC of virtual implant	14.85 ± 2.78	12.11 ± 0.78	0.0012

^a^Mann–Whitney U test.

## Discussion

The zygomatic bone is a dense, non-uniform structure with variable thickness, influenced by factors such as ethnicity and sex. In this study, the greatest thickness in the anterior, middle, and posterior regions was recorded at Point A1 (8.53 ± 1.63 mm), Point B_1_ (6.97 ± 1.01 mm), and Point C_0_ (6.36 ± 1.02 mm), respectively (Fig. 1). Overall, the zygomatic bone dimensions in this study were larger than those reported by Takamaru [15], Nkenke [16], and Xu [17], but comparable to the findings of Hung [13] and Pu [18] (Table 3). Consistent with Hung [13] and Nkenke [16], our results showed significantly greater zygomatic bone thickness and length in males than in females. In contrast, Takamaru et al. [15] reported no sex-related differences, likely due to their small sample size (11 dry skulls) and population differences. Notably, most previous studies were conducted on dry skulls, whereas our measurements were obtained using CBCT imaging.

**Table 3:** Comparison of zygomatic bone length and thickness among different studies.

Author	Country	Year	Sample	Results
Takamaru [14]	Japan	2016	Dry skull	Thickness: 1.6 ± 0.5 mm–7.2 ± 1.7 mm Length: 18.2 ± 2.5 mm–23.1 ± 4.1 mm
Jensen [25]	India	1992	Dry skull	Thickness: 4.5 mm
Nkenke [15]	Germany	2003	Dry skull	Thickness: 7.60 ± 1.45 mm (female), 8.00 ± 2.26 mm (male) Length: 25.40 ± 2.64 mm (female), 24.93 ± 4.67 mm (male)
Rigolizzo [19]	Brazil	2005	Dry skull	Thickness: 2.8–6.5 mm
Pu [17]	China	2014	CBCT	Thickness: 11.01 ± 2.77 mm
Hung [12]	China	2017	CBCT	Thickness: 4.51–8.01 mm Length: 25.67–32.54 mm
Xu [16]	China	2017	Dry skull	Thickness: 20.4 ± 2.61 mm Length: 5.6 ± 1.28 mm
Pellegrino [24]	Italy	2020	CBCT	Length: 55.13 ± 9.42 mm (male), 51.84 ± 8.85 mm (female)
This study	Vietnam	2021	CBCT	Thickness: 3.05–11.1 mm Length: 20.9–33.95 mm

In this study, the zygomatic bone exhibited significantly greater thickness at most measured points and greater length at all lines in males compared to females (Fig. 2). While Hung et al. [13] reported no significant sex differences at Points A_0_, A_1_, and C_0_, our study identified C_0_ and C_1_ as not significantly different between sexes. Furthermore, when examining the correlation between zygomatic bone thickness and length in individuals above and below 60 years of age, no statistically significant differences were found. This aligns with the study by Hung et al. [13]. and Pu et al. [18]., suggesting that age-related changes in anatomical landmarks for zygomatic implant placement remain inconclusive.

These results emphasize ethnic variations in zygomatic bone morphology, which may influence surgical planning and implant placement strategies. Differences between studies could be attributed to varying landmark selection and measurement methods. Notably, many previous studies identified the thickest region of the zygomatic bone without determining whether this area corresponds to the highest BIC. To address this limitation, our study not only measured zygomatic bone thickness at predefined landmarks but also performed virtual implant placement and evaluated BIC values to identify optimal implant sites. This method provides clinically relevant guidance for selecting implant positions with maximum stability. Moreover, Rigollizo et al. suggested that in cases of insufficient zygomatic bone thickness, implant placement can be aided by digital navigation systems [19, 20]. Compared to conventional techniques, navigation-assisted implant placement significantly improves precision and safety [21, 22].

We found that the virtual BIC measurement ranges from 4.75 to 17.15 mm (Fig. 3). In the anterior region, the maximum recorded BIC measurement is approximately 17.15 mm, which is greater than the results reported by Hung [13], He [23], and Bertos [24], but comparable to those of Pellegrino [25] (Table 4). These variations highlight the importance of precise measurement techniques and consideration of patient-specific anatomy when planning zygomatic implant placement.

**Table 4:** Comparison of virtual implant BIC among different studies.

Author	Country	Year	Sample	Average BIC Value
Bertos [23]	Spain	2017	CBCT	16.95 ± 4.73 mm
Hung [12]	China	2017	CBCT	4.12 ± 1.83 mm–16.7 ± 4.18 mm
He [22]	China	2021	CBCT	Anterior teeth: 13.80 ± 3.74 mm Posterior teeth: 13.90 ± 2.81 mm
Pellegrino [24]	Italy	2020	CBCT	17.92 ± 6.92 mm
This study	Vietnam	2021	CBCT	4.75 mm–17.15 mm

BIC, determined by the length of the implant engaging the zygomatic bone, is critical for predicting osseointegration, implant stability, and long-term success. Greater BIC enhances implant stability and compensates for the poor bone quality of the maxilla. Moreover, connecting multiple zygomatic implants improves biomechanical stability and increases the likelihood of treatment success. While BIC in histological contexts reflects direct biological integration, our radiographic BIC represents the predicted surface engagement between the implant and zygomatic bone. This approach is crucial in assessing the feasibility and expected primary stability of implant placement, especially in complex anatomical areas like the zygomatic region where intraoperative adjustments may be limited. Identifying high-BIC zones helps clinicians choose optimal implant positions and trajectories, improving surgical accuracy, minimizing complications, and supporting immediate loading protocols. Previous studies by Takamaru et al. and Rigolizzo et al. suggested that the areas with the highest BIC coincide with the thickest regions of the zygomatic bone [15, 20]. However, we found that the thickest points—A_1_, B_1_, and C_0_—did not provide the highest BIC values. Instead, the optimal sites for maximum BIC were A_3_, B_2_, and C_2_ in the superior, middle, and inferior regions, respectively, none of which corresponded to the thickest regions of the zygomatic bone.

The ITF houses critical neurovascular structures, including the maxillary artery and its branches, the pterygoid venous plexus, and the mandibular nerve. Owing to the curvature of the zygomatic bone and the linear trajectory of zygomatic implants, distal apex placement poses a substantial risk of fossa intrusion. Rossi et al. advocate for modifying the implant insertion angle to mitigate this risk and preserve adjacent anatomical structures [26]. In our study, no cases of ITF penetration were observed when implants were placed in the superior region of the zygomatic bone. However, posterior sites—specifically B_2_, B_3_, C_1_, C_2_, and C_3_—demonstrated a higher incidence of intrusion. Intrusion depth and length increased progressively from the inferior to the superior region, consistent with the trend reported by Wang et al. [27]. These findings highlight the anatomical challenges of posterior implant trajectories.

Based on BIC analysis and anatomical safety, the most favorable implant apex positions were A_3_ (17.65 ± 2.24 mm) in the superior anterior region and B_1_ (13.34 ± 2.35 mm) in the middle posterior region. This suggests that the anterior-superior and mid-posterior regions of the zygomatic bone offer the best combination of implant stability and minimal complication risk. These data have direct clinical implications for preoperative planning, supporting the selection of optimal anatomical sites for quad zygomatic implant placement.

The use of virtual BIC mapping and intrusion risk simulation enhances surgical decision-making and reduces the likelihood of intraoperative complications. This emphasis on individualized trajectory planning aligns with both anatomical and clinical studies. For example, the study on donated bodies assessing malar bone dimensions for safe zygomatic implant placement reported comparable patterns of regional bone thickness and emphasized the risk of posterior trajectory encroachment into critical anatomical spaces [28]. Their anatomical data validate the importance of precise implant angulation and reinforce the relevance of preoperative virtual planning. Moreover, a randomized controlled trial by Esposito et al. [29] showed that modifications in surgical approach did not compromise implant survival, underscoring the value of individualized anatomical planning over rigid protocol adherence.

The significantly greater length and thickness of the zygomatic bone, along with the significantly higher BIC of virtual implants in males compared to females at most points—particularly at Points A_3_ and B_1_ (Table 2)—suggest that quad zygomatic implants may achieve greater stability in males than in females. These sex-related anatomical differences have important implications for surgical planning, particularly in selecting implant length, diameter, and trajectory. Males, with greater bone volume, may accommodate longer implants or wider diameters, which can enhance biomechanical stability. Conversely, the comparatively thinner zygomatic bone in females may necessitate a more conservative approach, including the use of tapered or angled implants, shorter implant lengths, or adjunctive techniques such as bicortical engagement and navigation-assisted placement. Tailoring implant strategies to individual anatomical profiles may optimize clinical outcomes and minimize the risk of intraoperative complications, reinforcing the value of sex-specific planning in zygomatic implantology.

Unlike previous studies that primarily focused on the thickest points of the zygomatic bone, our study evaluated both bone thickness and BIC values at standardized anatomical landmarks. This method provides valuable clinical guidance for determining optimal implant placement and enhances preoperative planning. Furthermore, recognizing sex-related anatomical differences supports better case selection and prognosis in zygomatic implant treatment.

To our knowledge, this is the first study to comprehensively compare BIC values between males and females and to investigate optimal implant sites for quad zygomatic implant placement in the Vietnamese population. However, several limitations should be acknowledged. The exclusive inclusion of Vietnamese patients may limit the generalizability of our findings to other ethnic groups, as anatomical variations across populations can influence implant planning. Additionally, the relatively small sample size reduces the statistical power and may affect the robustness of the conclusions. Future studies with larger, more diverse cohorts are needed to validate these results and enhance their applicability. Furthermore, biomechanical simulations under functional loading conditions are recommended to assess stress distribution and implant performance. Longitudinal clinical studies correlating radiographic BIC estimates with actual implant outcomes would also strengthen the clinical relevance of virtual planning methodologies.

## Conclusions

Our study identified Point A_3_ in the anterior region and Point B_1_ in the posterior region as the most suitable sites for zygomatic implant placement, as these sites maximize BIC while minimize the risk of intrusion-related complications. Additionally, quad zygomatic implants may achieve greater stability in males than in females due to increased zygomatic bone thickness and higher BIC.

## Supplementary information

Figure S1 and Figure S2

## Data Availability

The data support the findings of this study are available upon reasonable request.
