# Supplementary material for: Optimal bone-implant contact sites in the zygomatic region for quad zygomatic implants placement: a retrospective study in Vietnamese patients on CBCT
Source: BDJ Open. 2025 Jun 20;11:61. doi: 10.1038/s41405-025-00350-8 (PMC12181228; doi:10.1038/s41405-025-00350-8)
Supplement: Supplementary file 1 — Figure S1 and Figure S2 [file 41405_2025_350_MOESM1_ESM.docx]

**** **SUPPLEMENTARY INFORMATION**

**Fig. S1.** Anatomical landmarks for zygomatic bone measurements.

1. The lines IM and LM; and the intersection points C and O
2. The line L_1_ connecting Point C and Point O
3. The lines L_0_ – L_3_ and the points A_0_ – A_3_, B_0_ – B_3_, and C_0_ – C_3_
4. The points E_1_ and E_2_

**
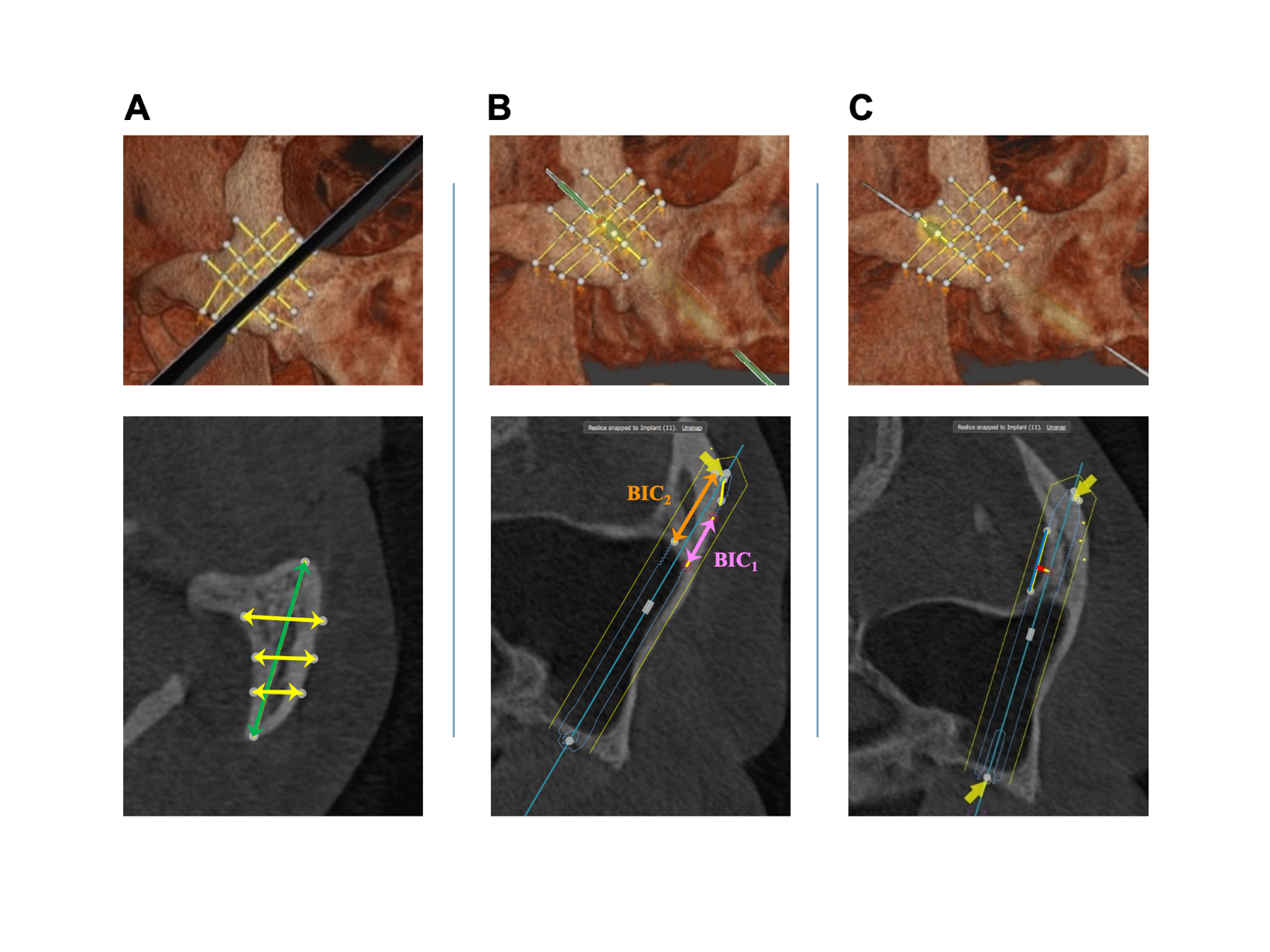
Fig. S2.** Longitudinal tomography showing the measurements of zygomatic bone length and thickness, bone-to-implant contact (BIC), and intrusive depth into infratemporal fossa (ITF)

A. Zygomatic bone length (green arrow) and thickness (yellow arrows)

B. The BIC length on the facial side (BIC_1_, pink arrow) and temporal side (BIC_2_, orange arrow)

C. The intrusive depth of implant into ITF (red line)
